# Supplementary material for: Precise determination of molecular adsorption geometries by room temperature non-contact atomic force microscopy
Source: Commun Chem. 2024 Jan 6;7:8. doi: 10.1038/s42004-023-01093-z (PMC10771516; doi:10.1038/s42004-023-01093-z)
Supplement: Supplementary file 2 — Supplementary Information [file 42004_2023_1093_MOESM2_ESM.pdf]

Supplementary Information

Timothy Brown*[1], Philip James Blowey[1], and Adam Sweetman*[1]

[1]The University of Leeds, Leeds, United Kingdom
*Corresponding authors: Timothy Brown, T.J.Brownphy@leeds.ac.uk, Adam Sweetman, A.M.Sweetman@leeds.ac.uk

Supplementary Note 1

Custom LabVIEW scripts were used to acquire the 3D data sets. periodically using atom tracking to measure and compensate for the thermal drift. The 3D Δf data sets were acquired via two principal methods, stacking images of constant height [1, 2], and grid spectroscopy [3, 4]. Supp. Fig. 1 a depicts the procedure for gathering constant height images iteratively, over the same region of the sample, while varying the tip-sample distance, z. Using the method described by Sugimoto et al. (2010) [1], the script engages tracking mode between scans over a desired site, in order to reverse drift-induced displacement and update the feedforward correction. The scan speed must be sufficiently fast to allow the tip to always return to the reference feature being tracked (an atom or a molecule).

Conversely, the grid spectroscopy method collates single-point $\Delta f(z)$ spectra, which collectively comprise a grid in the (x, y) plane as shown in Supp. Fig. 1 b ([3–5]). As with the constant height slices method, acquisition of the multi-dimensional data set via grid spectroscopy is regularly interrupted in order to compensate the thermal drift. Supp. Fig. 1 depicts how single $\Delta f(z)$ spectra can be extracted from the data cubes for analysis and force inversion (plotted in c)).

Conventionally, feedforward correction vectors are calculated via a linear fit of a continuous displacement of a feature the tip is locked on to. To achieve acceptable accuracy for the calculated linear fit, the tip should track the feature for ideally ~ 1 minute [6, 7]. Doing so significantly increases the duration of the experiment, and can be prone to errors when the thermal drift behaves non-linearly. Alternatively, the thermal drift can be calculated by measuring the change in position of a tracked feature before and after a period of data acquisition (e.g. a constant height scan). When in tracking mode, the LabVIEW script continuously measures the x, y and z positions of the tip over a short period of time (~ 10 seconds) whilst it dithers around a hill-like feature with feedback applied, and an average position p_0, is calculated. It is not necessary to leave the tip in tracking any longer than is needed to estimate just the position of the feature apex. Tracking mode is then disengaged, and data can again be acquired. Following this, the tip is brought back to p_0 and tracking is re-engaged. The position of the tracked feature is likely to have displaced slightly due to thermal drift, so the tip (via the lateral feedback mechanism) must spend some duration

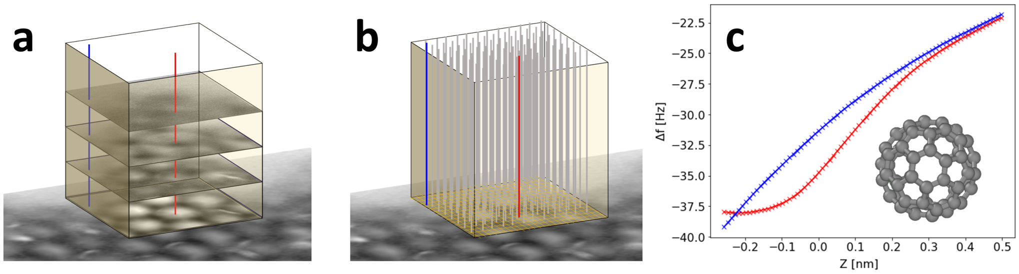

Supplementary Figure 1: Schematic for 3D data acquisition methods using an area of a C_{60} island as an example. a) Stacking constant height images in z. b) Compiling single point spectra in a grid. c) From each method, theoretically equivalent $\Delta f(z)$ curves ($\times$ data points for constant height slices, with grid spectroscopy represented by continuous lines) can be extracted for force inversion. A red on curve and a blue off curve are plotted as examples, with positions shown in a) & b) also. Insert: ball-and-stick model of C_{60}.

of time to find the apex. The new position is reached p_1, and a set of feedforward correction vectors (V_x, V_y, V_z) is calculated from the difference $p_1 - p_0$, divided by the time taken between tracking measurements $t_1 - t_0$. These feedforward correction vectors V_x, V_y and V_z, are applied to the piezo scanner via the Nanonis controller. Subsequent measurements would then be conducted with a more accurate compensation of the thermal drift, and the process is continually repeated.

In addition to regularly updating the feedforward vectors, it is also necessary to compensate for the displacement $p_1 - p_0$. This vector is applied by the LabVIEW script, via the Nanonis controller, to the (x, y) coordinates of the next scan frame (or next point spectrum position). Displacement in z due to thermal drift is already corrected by virtue of feedback operation during tracking. The latter position, p_1 of one measurement, becomes the former position, p_0 of the next measurement. Supp. Fig. 2 depicts a timing diagram, for constant height data acquisition in SPM, with the LabVIEW drift correction protocol. To mitigate the risk of tip-changes in the absence of feedback, it was necessary to keep the tip high above the surface during lateral tip movements across the sample (moving to and from the tracking position).

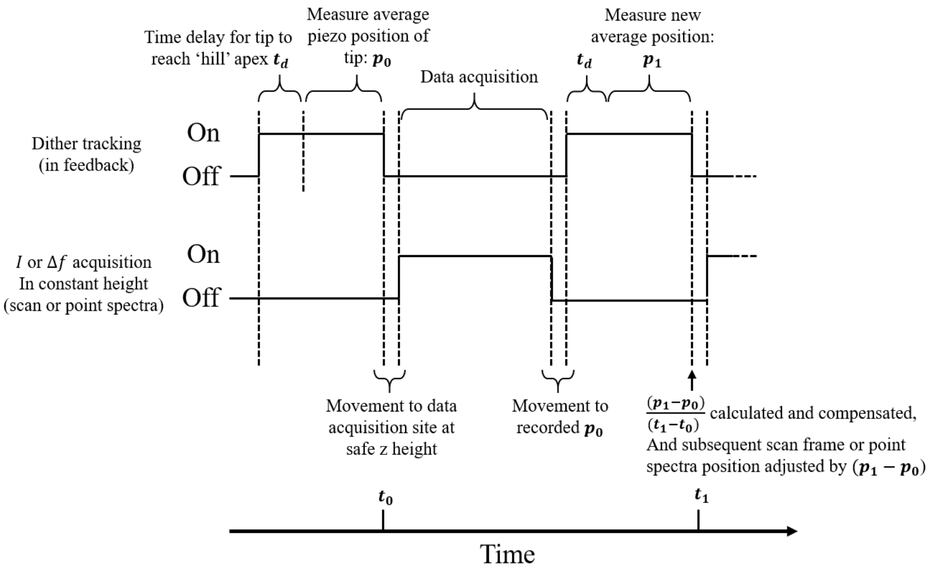

Supplementary Figure 2: Timing diagram for acquisition of large multi-dimensional data sets without feedback in either STM or NC-AFM. Starting from tracking mode, an average piezo position of the tip p_0, is measured after waiting sufficient time, t_d for the tip position to stabilise. The dither signal and feedback are then both disengaged. The tip then moves in constant height to its intended lateral position (with an offset applied to P_z to ensure a safe distance is maintained whilst in transit). Next, the I and / or Δf signals are recorded whilst conducting a scan, or point spectrum. The constant height data measured will be relative to the z component of p_0. Afterwards, the tip is moved back to p_0, and the feedback and tracking electronics are re-engaged. The position of the tracking apex (or hill) will have changed slightly due to residual thermal drift. A duration of time (t_d) is waited to allow the tip to stabilise at the new position, p_1 following a measurement of the average position of the tip. The vector $p_1 - p_0$ is calculated at that moment and then applied as an offset to the next intended position of the tip, helping to reverse the displacement. By recording the time when the position is measured, the current velocity of the drift can be estimated. This velocity is applied to the piezo as a feedforward correction, such that the DC signal sent to piezo scanner has an up to date compensation vector through the subsequent data acquisition. This process iterates as more elements of the data set are acquired.

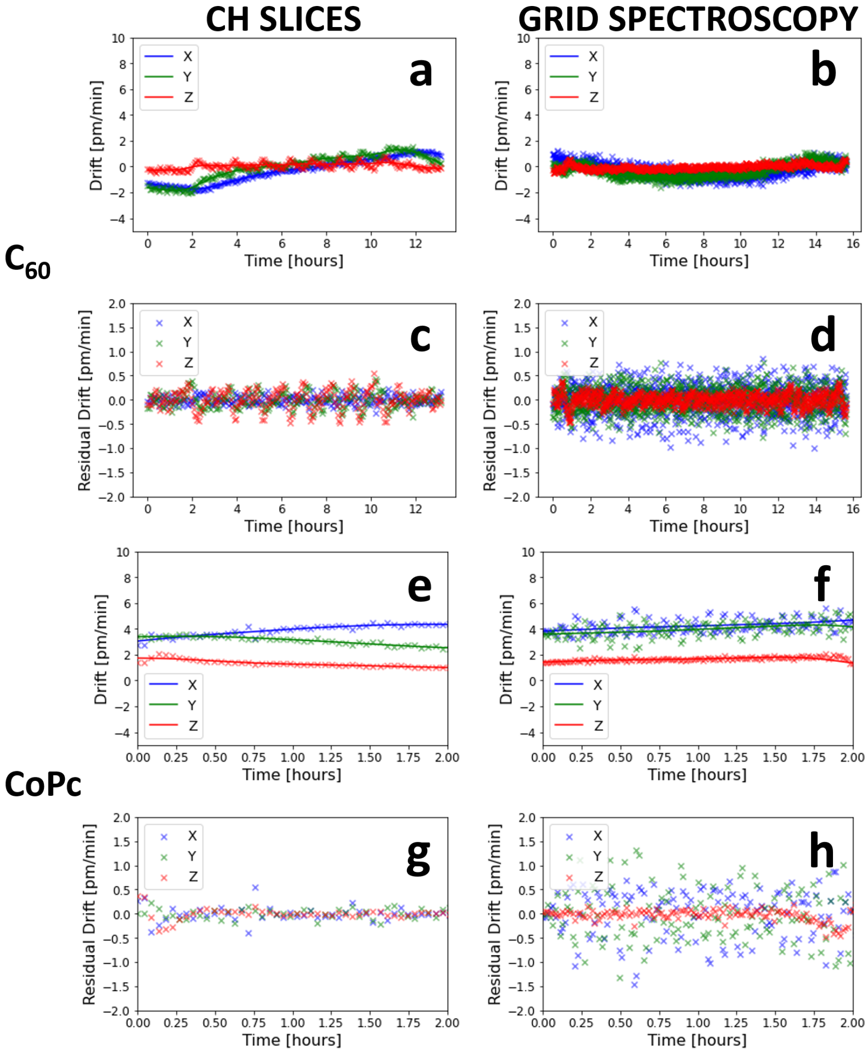

Supplementary Figure 3: Feedforward corrections and residual drifts calculated during $\Delta f(x, y, z)$ mapping experiments, using both methods of data acquisition: constant height slices and grid spectroscopy. a) & c) Applied feedforward vectors and calculated residual drifts (respectively) for a constant height slice map over C_{60} molecules (main result in main text Fig. 5). b) & d) Applied feedforward vectors and calculated residual drifts for a grid spectroscopy map over C_{60} molecules (main result Fig. 6 of main text). e) & g) Applied feedforward vectors and calculated residual drifts for a constant height slice map over CoPc molecules (main result in Fig. 3 of main text). f) & h) Applied feedforward vectors and calculated residual drifts for a grid spectroscopy map over CoPc molecules (main result not presented). For all experiments the temperature of the microscope head was regulated to at least ± 4 mK. A smooth spline was fitted to the data to estimate the true drift velocity of the system. Estimates of the residual drift of the system were calculated by subtracting the fitted splines from the measured drift vectors.

The atom tracking and scripting rely on the tip being able to reliably return to the same surface feature at each interval in the experimental measurement. This requirement poses an upper limit to the acquisition time t between the times where the tip is in feedback and

tracking mode:

$$r_{\text{drift}}(t) - r_{\text{drift}}(0) < \Delta g \tag{1}$$

Where Δg is the desired precision of the lateral positioning [4]. The data in Supp. Fig. 3, depicts the feedforward corrective vectors applied to the scan-tube, and hence gives an approximation of the thermal drift between the tip and sample. The variation in drift is reduced by regulating the system temperature. Without regulation, the drift can vary on the order of 100 $pm\ min^{-1}$ between each update of feedforward vectors, whereas the data across Supp. Fig. 3, exhibit variations less than 1 $pm\ min^{-1}$ between each tracking event. The drift correction of the slice method exhibited better performance over the grid spectroscopy method, despite requiring larger acquisition times between tracking. As these experiments were carried out consecutively (for each respective molecule), it is unlikely the external environment was the reason for the difference in thermal drift values. A possible explanation for the poorer performance of the grid spectroscopy could be the piezo-creep. After moving distances of the order of 1 nm in z, the tip resumes feedback and tracking, and the piezo crystal may be still relaxing during the measurement of its average position. It is notable that Rahe et al. (2011) [4] reported the use of feedforward correction with grid spectroscopy, yielding a maximum residual drift velocity of 25 $pm\ min^{-1}$ for an experiment that updated the feedforward correction after intervals of $\sim$ 15 minutes. In their experiment, the tip was re-centred on the point of tracking after intervals of 18.5 s in order to keep the displacement due to thermal drift under the desired precision / pixel density. Thus, $25 \times (18.5/60) \sim 8\ pm$ maximum displacement due to residual drift. This satisfies Supplementary Equation 1: $8\ pm < \Delta g \sim 50\ pm$, despite the lengthy durations between updates to the feedforward vectors. Similarly, the residual drift vectors presented in Supp. Fig. 3 also all satisfy the constraint within Supplementary Equation 1, for the respective pixel densities. Therefore the residual thermal drift is not a limiting factor of the lateral precision throughout the experiments in this paper (likely the limiting factor is the tip state). From examining the contrast and size of the intramolecular features, we estimate the lateral resolution of our system to be $\sim$ 50 pm, typical for room temperature measurement.

To conclude, both methods are capable of gathering high resolution force map data sets at room temperature with positional errors of the order 1 pm. The better performance in lateral stability of the slices experiment is insignificant when compared to the size of dither radius used. However, empirically, grid spectroscopy experiments (particularly when attempted in 3D), are more prone to unexpected tip-changes. This is likely because the tip reaches the point of closest approach for each pixel in (x, y).

Supplementary Note 2

Overview images were imported into Gwyddion software [8] and processed to make the figures in the main text. The other data (Nanonis .sxm files and spectroscopy data) were

imported into, and analysed using, custom-written Python code [9].

We observed a slow, systematic drift, in Δf, of the long-range interaction throughout the days-long experiment presented in the main text (Fig. 4), most likely owing to a drift in the resonance frequency, f_0 of the cantilever. We believe this to be a result of thermal fluctuation of the cantilever, or the reference oscillation of the PLL [10]. The data cubes were shifted in Δf to align the long range elements of the curves with that of the last mapping experiment. This was done using a linear regression to minimise the residual Δf shift between subsequent curves in the sequence, as was done for our previous room temperature force mapping experiments [11].

For the data sets of constant height images, each height was scanned three times and averaged to improve the signal-to-noise (S/N) ratio. No $post - hoc$ alignment of the constant height images was carried out across our data, as image alignment was already sufficient due to feedforward compensation. The forward and backward channels of the raster scans and point $\Delta f(z)$ spectra were averaged, having checked for any discrepancies between them.

For inversion into force data, the background signal (composed of only long range interactions) was removed by subtracting $\Delta f(z)$ curves taken over the bare substrate, within the data cubes. This is also known as an off-curve [12, 13] method of force inversion. The short range Δf signal created was then converted into the vertical tip-sample force using an algorithmic form of the Sader-Jarvis method [14, 15]. Before conversion, the $\Delta f(z)$ curves were smoothed using the csaps python library. The smoothing parameter, λ, defines the level of data smoothing from $\lambda = 0$ being a least squares linear fit through the data, and $\lambda = 1$ being the natural cubic spline interpolant [16]. The best value of λ was determined by eye for each data set (whilst ensuring the curves were not over-smoothed). In general this would be $\lambda \sim 0.99 - 0.999$. Following force inversion, the calculated $F(z)$ curves were also spline smoothed. Lateral averaging across pixel windows $\sim 150 \times 150\ pm^2$ in size were applied for the images, and $\Delta f(z)$ & $F(z)$ curves extracted in the main text figures.

The topographies of the on-set of repulsive interaction for each point in (x, y) over the CoPc molecules, were determined using Supplementary Equation 2:

$$z^*_{\Delta f}(x, y) = \arg\ \min \Delta f_z(x, y, z) \tag{2}$$

The $z^*_{\Delta f}(x, y)$ maps out the positions in z associated with the turnaround point in the Δf signal ([17, 18]). Before $z^*_{\Delta f}(x, y)$ extraction, the Δf images were smoothed with a rolling average in the (x, y) plane with a window size of $\sim 150 \times 150\ pm^2$. The Δf data was then smoothed in the z direction using a spline fit at each point in (x, y) ($\lambda = 0.99$). The magnitude of the $\Delta f(z)$ at each turnaround point across (x, y) was also extracted, also known as a $\Delta f^*(x, y)$ map. Given the apparent tip relaxation at closer tip-sample distances, the $\Delta f(z)$ signal becomes complex in both CoPc experiments (Figures 2 & 3 in the main text). Supp. Fig. 4 depicts how this complex behaviour in the Δf signal manifests itself in

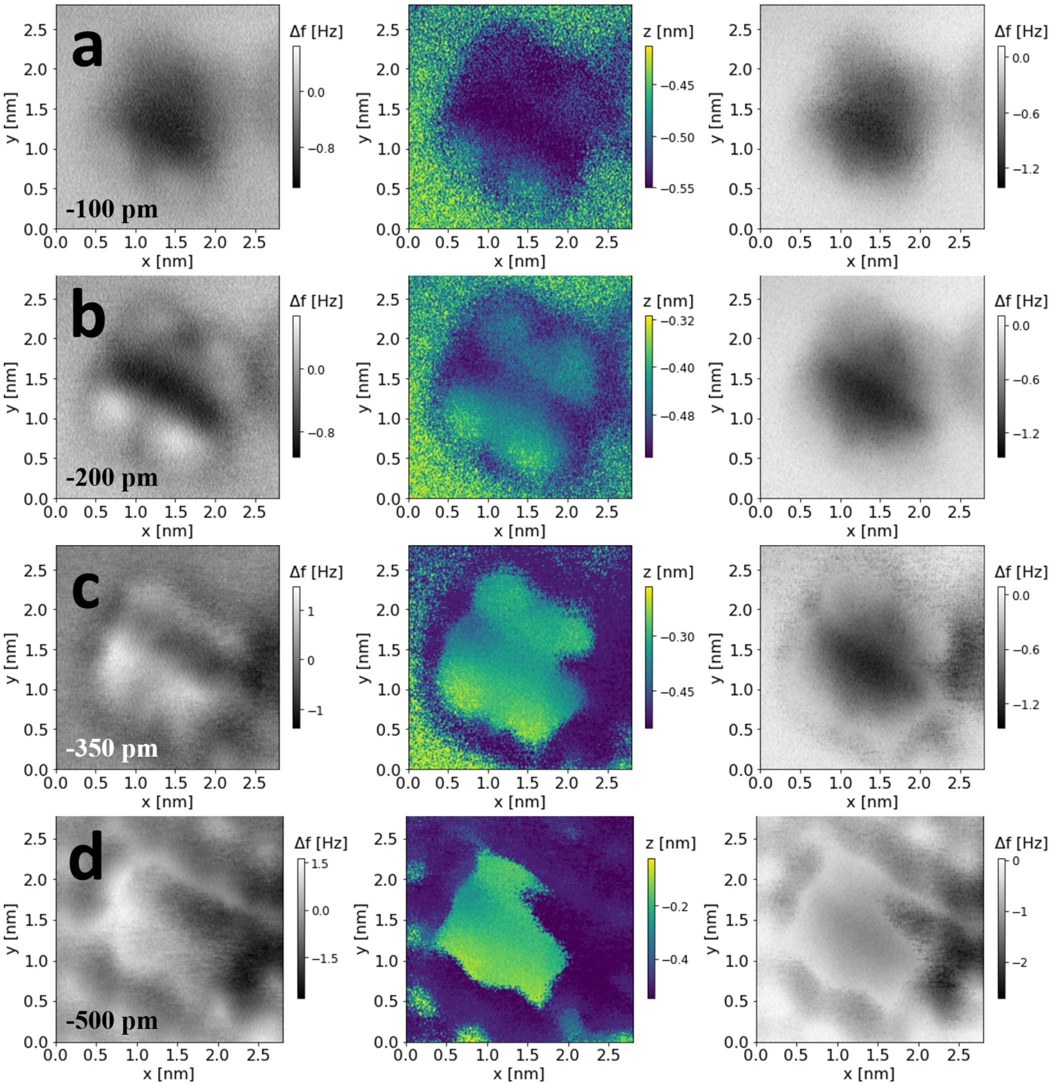

Supplementary Figure 4: Constant height Δf images (left), z^* (middle) and Δf^* maps (right) extracted from a Δf cube of a single CoPc (main result of which is presented in Fig. 2 in the main text). The z^* and Δf^* images are calculated using a portion of the full cube, from the top slice, to the heights associated with the corresponding Δf image (a) $= -100\ pm$, b) $= -200\ pm$, c) $= -350\ pm$, d) $= -500\ pm$).

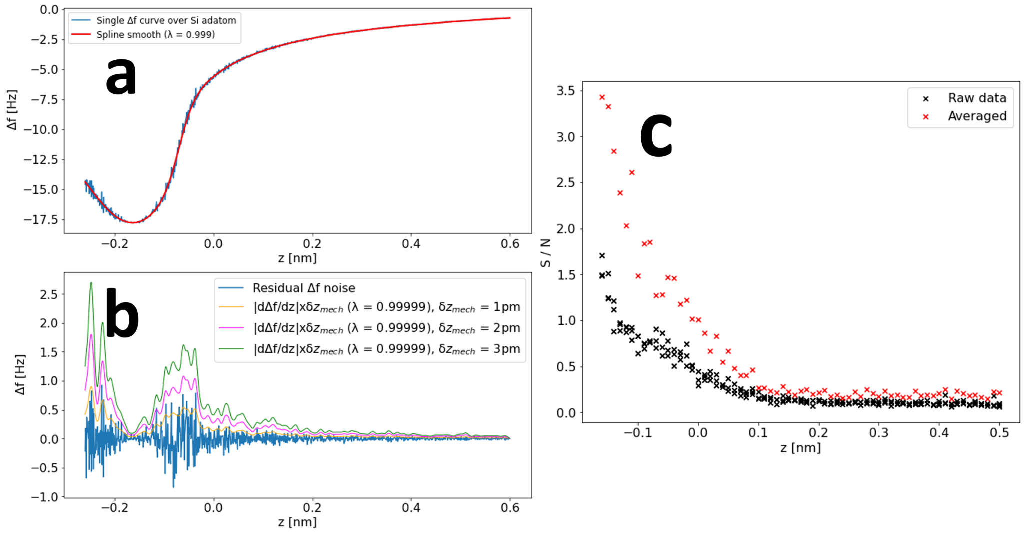

Supplementary Figure 5: a) Single point $\Delta f(z)$ curve taken over a Si(111)-(7×7) adatom. A spline fit curve ($\lambda = 0.99$) is plotted to estimate the Δf noise. b) Measured Δf noise (the differences between the raw $\Delta f(z)$ signal and the spline fit) for curve a). Models of the mechanical noise were plotted using the method proposed by Sugimoto et al. (2010) [1] in order to estimate the value of δz_{mech} that best fits the experimental data. The raw output of Supplementary Equation 3 was smoothed slightly for clarity via spline fitting ($\lambda = 0.9999$). c) Δf noise levels (as S/N) in slices-based $\Delta f(x, y, z)$ mapping experiments conducted over a bare Si(111)-(7×7) substrate. The S/N was calculated for both raw data and averaged images (of 3 scans) in Python using a FFT filtration to extract the signal image, from the noise.

both the extracted z^* map, and map of the minimum values of Δf. The images on the left depict representative constant height images, at varying height, from the data cube used to produce Fig. 2 in the main text. In the middle and right columns are z^* & Δf^* maps respectively, calculated using the data from the top slice, to the height associated with the corresponding scan on the left. The bottom row depicts the lowest constant height image, and therefore the z^* & Δf^* maps express the output of Supplementary Equation 2 when using the full span in z of the data cube. Across all three sub figures there are artefacts, whose origins are likely the result of complex relaxations in the tip-sample junction at close approach, due to the non-ideal termination of the tip, relaying abnormal $\Delta f(z)$ curves, e.g. exhibiting more than one Δf minima. As a result, the extraction of the z^* maps was modified to ensure that for each point in (x, y), only the position of the first $\Delta f(z)$ turnaround was extracted and plotted. Points in (x, y) that do not have a $\Delta f(z)$ turnaround were also screened. The resultant image, using the data in Supp. Fig. 4, is presented in Fig. 2.E in the main text.

Supplementary Note 3

From a thermal measurement of our commercial instrument [19], we estimate a high deflection noise of 600 $fm\ Hz^{-1/2}$, which is in fact inferior to both the optical interferometer setup and an optimised qPlus sensor at room temperature (reported as 15 $fm\ Hz^{-1/2}$ and 60 $fm\ Hz^{-1/2}$ respectively, in [20]). It is also inferior to a commercial low temperature

qPlus setup measured in our own laboratory (approx. 200 $fm\ Hz^{-1/2}$). It is noteworthy that the relatively high detector noise of our commercial room temperature instrument did not inhibit our ability to obtain high-resolution intramolecular contrast, or high-resolution force maps. As we note in the main text, we estimate the most significant source of noise in our measurement was the z stability of our microscope (excluding limited measurement time due to thermal stability as a factor).

The Δf noise of our system was characterised prior to the experiments using a point $\Delta f(z)$ spectrum conducted on silicon adatoms of a clean Si(111)-(7×7) surface, depicted in Supp. Fig. 5. A spline fit was used to assess the magnitude of noise. For both curves, there is a reduction in noise at the $\Delta f(z)$ turnaround. This indicates the dominant source of noise at close approach in the NC-AFM experiments was the mechanical noise, δz_{mech} of the microscope, since the noise contribution from δz_{mech} is proportional to the slope of Δf [1].

The raw error signal in $\Delta f(z)$ from a) is plotted in b) (blue). Using the methodology proposed by Sugimoto et al. (2010) [1]), calculated models of the mechanical noise of the system were also plotted, for various values of δz_{mech}:

$$\Delta f_{noise} = |\frac{d\Delta f}{dz}| \times \delta z_{\text{mech}} \tag{3}$$

δz_{mech} values of 1 pm, provided the best fit. Thus, the dominant source of noise in the system is mechanical, and the magnitude of that mechanical noise can be estimated as 1 pm. Using a 3D data cube gathered via constant height imaging of the silicon adatoms, the evolution across z of the S/N ratio was plotted for both raw scans and averaged images (of 3 scans) in Supp. Fig. 5 c). For the raw data at far distances, the S/N approaches zero, and at the point of closest approach, S/N $\sim 1.5 - 2$. Averaging the image over 3 scans increases the S/N ratio by approximately a factor of 2.

Supplementary Note 4

We include here in Supp. Fig. 6 an example of the dissipation and amplitude channels during the C_{60} force mapping experiments. The dissipation image has been converted from the excitation channel (units of (V)) into the energy dissipation of the approach and retraction of the tip (units of (meV / cycle)) using the equation for energy dissipation arising from the tip-sample interaction [10]:

$$\Delta E_{ts} \ = \ \frac{2\pi E_0}{Q}(\frac{A'_{drive}}{A_{drive}} \ - \ 1) \tag{4}$$

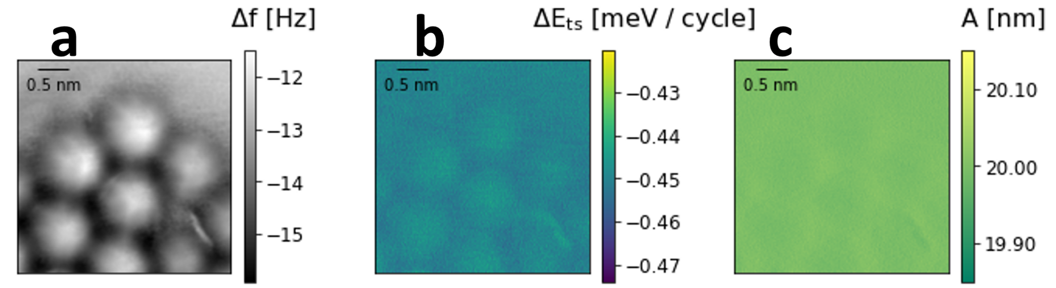

Supplementary Figure 6: a) Raw Δf, b) dissipation, and c) amplitude channels of the scan of closest approach from a C_{60} force map experiment (was result present in Fig. 5 in the main text).

Where E_0 is the energy stored in the motion of the cantilever ($\frac{k}{2}A_0^2$), A'_{drive} is the driving amplitude measured at the lowest slice, and A_{drive} is taken to be the free damping signal, from the top slice. The dissipation and driving amplitude signals are negligible. This is likely owing to a predominantly conservative interaction between the sample and a passivated tip (similar to measurements with CO tips on molecules using a qPlus sensor at low temperature), and thus do not affect our force reconstruction.

Supplementary Note 5

In previous experiments, the organic molecule naphthalene tetracarboxylic diimide (NTCDI) has shown to be amenable to force mapping at room temperature [11]. In addition, ordered networks of NTCDI, mediated by H-bonding interactions have previously been successfully prepared on a passivated semiconducting surface (Ag:Si(111) $-(\sqrt{3}\times\sqrt{3})R30°$) at 77 K [2]. As such, NTCDI was explored as a possible candidate to apply submolecular force mapping experiments over a network of molecules assembled on the B:Si(111) $-(\sqrt{3}\times\sqrt{3})R30°$ surface. For a partial coverage ($\sim 0.2ML$), NTCDI was deposited at 175$°C$ for 2 hours onto a room temeprature substrate, however the NTCDI was not observed to form ordered islands. This was true for a range of deposition rates (e.g. 190$°C$ for 5 minutes), and also after annealing the sample at 200$°C$ (for 30 minutes). Supp. Fig. 7 a & b show both STM and NC-AFM overviews of disordered NTCDI islands on B:Si(111). a) The STM reveals that the majority of the surface defects are covered by the NTCDI, and furthermore that the coverage of NTCDI ($10-20$ %) is greater than the defect density. The NTCDI bonds to both the dangling bond defects, and the boron substituted sites of the surface. b) Reveals intramolecular features, not dissimilar from the contrast observed for adaptive height overviews of NTCDI on Si(111)-(7×7) [11]. However, unlike for the Si(111)-(7×7) surface, contrast matching the expected chemical structure of NTCDI was not observed. Following this, a deposition (200$°C$ for 30 minutes onto a room temperature substrate) was attempted to see if coverage approaching a monolayer would produce an ordered formation. Similarly, no ordered structures were observed. A possible explanation is that the oxygen atom within the carbonyl group still interacts strongly with the passivated silicon atoms, causing them to pin to the surface. By extension, it is believed that other molecules that include caronbyl groups (ketones, aldehydes, acids), in addition to molecules bearing OH groups (alcohols, phenols), would pin to the B:Si(111) surface in a similar way.

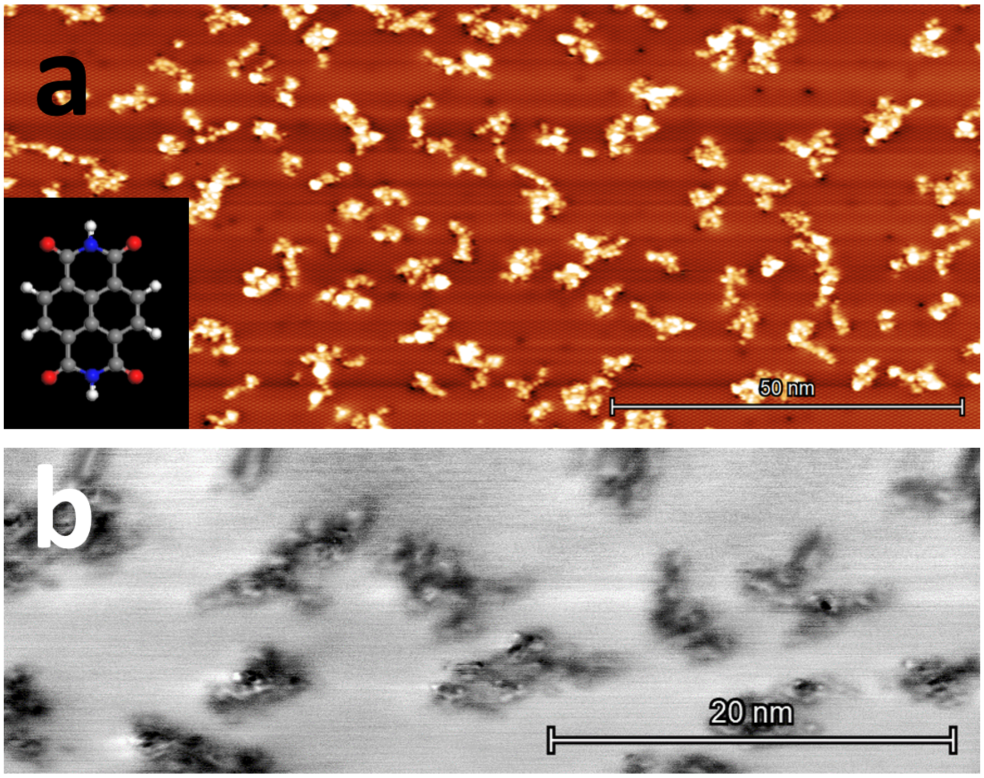

Supplementary Figure 7: ([11, 21]) Overview images of disordered NTCDI island formation on B:Si(111). a) STM constant current image. Image parameters: $V_{gap} = +2\ V$ and set point = 20 pA. The insert depicts a ball-and-stick model of a NTCDI molecule. b) NC-AFM image scanned in adaptive height (or pseudo-constant height) mode [11, 21]). Image parameters: $V_{gap} = 0\ V$, Oscillation amplitude 22 nm, set point = $-6\ Hz$, adaptive height offset = $-270\ pm$.

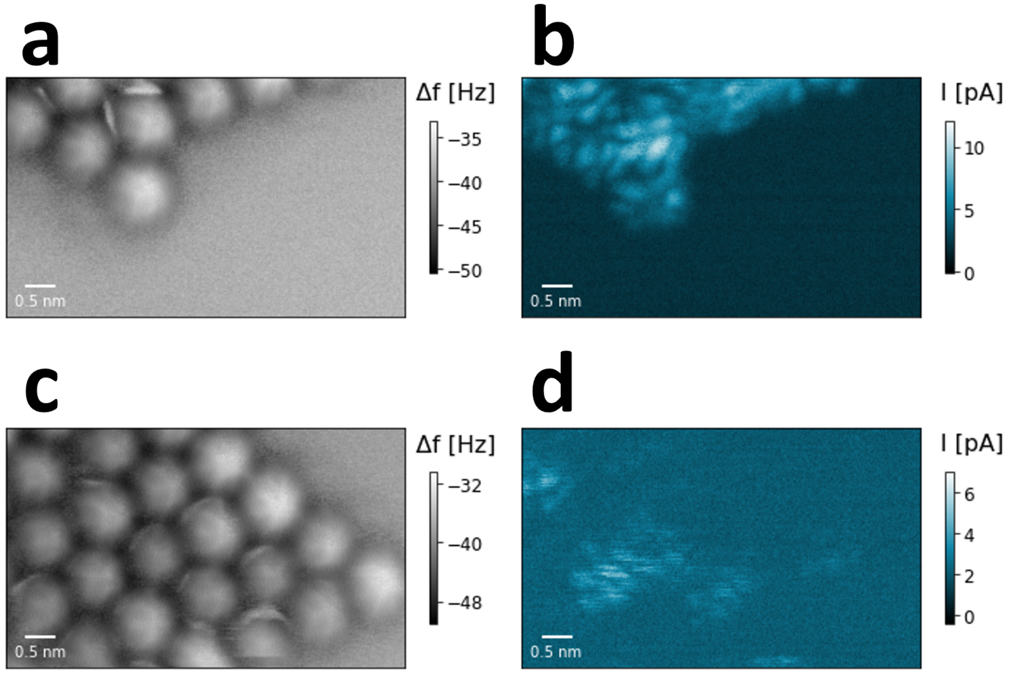

Supplementary Figure 8: a) Δf signal of the lowest scan in Δf map #3 (see Fig. 4 in main text). b) Corresponding current channel. c) Δf signal of the lowest scan in Δf map #4. d) Corresponding current channel.

Supplementary Note 6

Supp. Fig. 8 b & d shows example images of the tunnel current signal measured across the constant height scans, from the third of fourth maps of the continuous Δf mapping experiment (see Fig. 4 of the main text). The tunnel current in b) in particular, exhibits typical lobe features of constant height STM images of C_{60}. Whether or not there was a detectable tunnel current during NC-AFM was dependent on the tip state, but when there was, it was small in magnitude ($\leq 10\ pA$). Throughout the data presented in this paper, only the first three of the five Δf maps presented exhibit a consistent tunnel current reading over the C_{60} molecules. This would indicate, that despite there being negligible change in the force minima and image contrast, the tip state changed at some point between the point of closest approach of map #3 and #4 (having already established a change between #2 and #3 by looking at the Δf behaviour). From the image data throughout map #4, it is not possible to determine a more precise time of this tip change.

Supplementary References

(1) Sugimoto, Y.; Nakajima, Y.; Sawada, D.; Morita, K.-i.; Abe, M.; Morita, S. *Physical Review B* **2010**, *81*, 245322.

(2) Sweetman, A. M.; Jarvis, S. P.; Sang, H.; Lekkas, I.; Rahe, P.; Wang, Y.; Wang, J.; Champness, N. R.; Kantorovich, L.; Moriarty, P. *Nature Communications* **2014**, *5*, 3931.

(3) Fremy, S.; Kawai, S.; Pawlak, R.; Glatzel, T.; Baratoff, A.; Meyer, E. *Nanotechnology* **2012**, *23*.

(4) Rahe, P.; Schtte, J.; Schniederberend, W.; Reichling, M.; Abe, M.; Sugimoto, Y.; Khnle, A. *Review of Scientific Instruments* **2011**, *82*.

(5) Kawai, S.; Glatzel, T.; Koch, S.; Baratoff, A.; Meyer, E. *Physical Review B - Condensed Matter and Materials Physics* **2011**, *83*, 1–7.

(6) Abe, M.; Sugimoto, Y.; Custance, O.; Morita, S. *Applied Physics Letters* **2005**, *87*, 173503.

(7) Sugimoto, Y.; Jelínek, P.; Pou, P.; Abe, M.; Morita, S.; Pérez, R.; Custance, Ó. *Physical review letters* **2007**, *98*, 106104.

(8) Nečas, D.; Klapetek, P. *Central European Journal of Physics* **2012**, *10*, 181–188.

(9) Van Rossum, G.; Drake Jr, F. L., *Python reference manual*; Centrum voor Wiskunde en Informatica Amsterdam: 1995.

(10) Giessibl, F. J. *Reviews of Modern Physics* **2003**, *75*, 949–983.

(11) Brown, T.; Blowey, P. J.; Henry, J.; Sweetman, A. *ACS Nano* **2023**, *17*, 1298–1304.

(12) Ternes, M.; González, C.; Lutz, C. P.; Hapala, P.; Giessibl, F. J.; Jel inek; Heinrich, A. J. *Phys. Rev. Lett* **2011**, *106*, 16802.

(13) Sweetman, A.; Stannard, A. *Beilstein Journal of Nanotechnology* **2014**, *5*, 386–393.

(14) Sader, J.; Jarvis S, P. *Appl. Phys. Lett.* **2004**, *84*, 1801–1803.

(15) Stannard, A.; Sweetman, A. M., *A Considered Approach to Force Extraction from Dynamic Force Microscopy Measurements*; Moriarty, P., Gauthier, S., Eds.; Springer International Publishing: 2015, pp 63–79.

(16) De Boor, C., *A Practical Guide to Splines*; Springer New York, NY: 1978.

(17) Mohn, F.; Gross, L.; Meyer, G. *Applied Physics Letters* **2011**, *99*, 53106.

(18) Gross, L.; Schuler, B.; Liu, W.; Tkatchenko, A.; Moll, N.; Meyer, G.; Mistry, A.; Fox, D. *Physical Review Letter* **2013**, *111*, 106103.

(19) Lubbe, J.; Temmen, M.; Rode, S.; Rahe, P.; Kuhnle, A.; Reichling, M. *Nanotechnol.* **2013**, *4*, 32–44.

(20) Iwata, K.; Yamazaki, S.; Mutombo, P.; Hapala, P.; Jelínek, P.; Sugimoto, Y. *Nature Communications* **2015**, *6*, 7766.

(21) Moreno, C.; Stetsovych, O.; Shimizu, T. K.; Custance, Ó. *Nano Letters* **2015**, *15*, 2257–2262.
