# Supplementary material for: Precise determination of molecular adsorption geometries by room temperature non-contact atomic force microscopy
Source: Commun Chem. 2024 Jan 6;7:8. doi: 10.1038/s42004-023-01093-z (PMC10771516; doi:10.1038/s42004-023-01093-z)
Supplement: Supplementary file 3 — Description of Additional Supplementary Files [file 42004_2023_1093_MOESM3_ESM.pdf]

Description of Additional Supplementary Files

File name: Supplementary Movie 1

Description: Video version of Figure 4.
